# A Retrospective Cohort Study to Optimize Piperacillin/Tazobactam Dosing in Routine Intensive-Care Patients via a Newly Designed Clinical Pharmacy Service Including TDM- and Algorithm-Based Recommendations

**DOI:** 10.3390/pharmacy14050120

**Published:** 2026-08-17

**Authors:** Johanna Dicken, Daniel Dönisch, Jan Hensen, Olaf Zube, Thilo Bertsche

**Affiliations:** 1 Pharmacy Department, Bundeswehr Hospital Hamburg, 22049 Hamburg, Germany; 2 Department of Anesthesia and Intensive-Care, Bundeswehr Hospital Hamburg, 22049 Hamburg, Germany; 3 Clinical Pharmacy Department, Institute of Pharmacy, Medical Faculty, Leipzig University, 04103 Leipzig, Germany; 4 Drug Safety Center, Medical Faculty, Leipzig University Hospital, Leipzig University, 04103 Leipzig, Germany

**Keywords:** piperacillin, therapeutic drug monitoring, drug dosage calculations, clinical-pharmacy-services, hospital pharmacy, pharmacy management, decision management

## Abstract

*Background:* Therapeutic drug monitoring (TDM) for piperacillin/tazobactam has become established in intensive care. TDM should be used in a structured approach to optimize dosing. We evaluated a newly designed TDM- and algorithm-based clinical-pharmacy-service to determine whether it is appropriate for optimization. *Methods:* We developed a clinical pharmacy service providing algorithmic recommendations for optimizing piperacillin/tazobactam dosing based on piperacillin TDM. After implementing the clinical pharmacy service, we evaluated the treatment of intensive-care patients with piperacillin/tazobactam in terms of achieving the minimum-inhibitory-concentration-(MIC)-related target concentration at the following four time points: t1, t2, t3, and t4 (1–4 working days after piperacillin/tazobactam therapy was started). *Results:* A total of 132 patients (including 36% women), median age 78 years, received piperacillin/tazobactam therapy. Sepsis or septic shock was the most common indication (43%), while a pulmonary focus was most frequent (40%). From 132 patients (t1), 19% had “too low piperacillin concentrations”, 20% “appropriate concentrations”, and 61% “too high concentrations”. After recommendations were given (acceptance rate 100%) the corresponding numbers from 93 remaining patients (t2) were 16%, 35%, and 48%, from 49 patients (t3) 8%, 53%, and 39% and from 29 patients (t4) 38%, 45%, and 17%. In (t1), dose reduction was the main recommendation (49%). The number of patients with “appropriate concentrations” increased in one comparison (t1–t2: n.s.; t1–t3: *p* = 0.0013; t1–t4: n.s.; Bonferroni-corrected McNemar test), while “too high concentrations” decreased (t1–t2: *p* = 0.0080; t1–t3: *p* = 0.0056; t1–t4: *p* = 0.0095). “too low concentrations” were not influenced (t1–t2: n.s.; t1–t3: n.s.; t1–t4: n.s.). *Conclusions:* Inappropriate piperacillin plasma concentrations were common. A TDM- and algorithm-based clinical-pharmacy-service especially decreased the number of overdosed patients in routine practice.

## 1. Introduction

Beta-lactam antibiotics are by far the most frequently prescribed group of antibiotics [1]. This is the case since β-lactam antibiotics demonstrate high and sufficiently broad efficacy in a calculated therapy against the most common expected pathogens and have good tolerability and low toxicity [2]. The fixed combination of piperacillin with the beta-lactamase-inhibitor tazobactam is a frequently used β-lactam antibiotic combination in calculated and targeted anti-infective therapy in intensive-care patients [3]. In this regard, piperacillin/tazobactam, administered in an 8:1 ratio, is reported to be an effective treatment for patients with lower respiratory tract, intra-abdominal, urinary tract, gynecological and skin/soft tissue infections, and for fever in patients with neutropenia [4]. Piperacillin/tazobactam is generally well-tolerated, with gastrointestinal symptoms (most commonly diarrhea) and skin reactions being the most frequently reported adverse drug reactions [4].

In recent years, variable and especially potentially suboptimal plasma concentrations in intensive-care patients have come into focus [5]. Since the antimicrobial effectiveness or pharmacokinetics/pharmacodynamics (PK/PD) of β-lactam antibiotics is defined by the time above the MIC (minimum inhibitory concentration, t > MIC), it is important that a threshold based on the MIC is exceeded for the therapy [6]. It follows that higher plasma concentrations do not necessarily lead to improved efficacy but, despite relatively good tolerability, may be associated with a higher rate of adverse drug reactions [2,6]. Due to its relatively good tolerability, it should be borne in mind that too low piperacillin plasma concentrations are generally considered more serious than too high concentrations. The clinical picture of infections, especially in intensive-care patients, is subject to high intraindividual dynamics, and patients exhibit highly variable pharmacokinetics between individuals, particularly with specific properties such as renal replacement therapy [7]. Additionally, sepsis-related physiological changes and medical interventions (e.g., mechanical ventilation, surgery, and vasopressors) can significantly alter antibiotic kinetics. Renal impairment may decrease drug clearance and prolong half-life, while fluctuating fluid balance makes renal function difficult to assess. Conversely, some patients experience increased renal clearance, especially affecting renally eliminated antibiotics, e.g., beta-lactams, potentially leading to subtherapeutic drug concentration [8]. As a consequence, rigid dosage regimens, as often included in the drug label (i.e., summary of product characteristics), are frequently not useful in routine care. All those factors, furthermore, contradict the need to achieve sufficiently high plasma concentrations as quickly as possible [9,10]. Additionally, other factors such as obesity and the MIC of the particular pathogen also have a high relevance for antibiotic dosage [11,12]. Therefore, therapeutic drug monitoring (TDM) has been recommended for the measurement of plasma concentrations in those patients [6,13,14,15,16]. TDM followed by tailored dose optimization helps more patients to achieve the predefined target values of piperacillin/tazobactam, resulting in improved efficacy and decreased toxicity [17]. On the other hand, undesirable dose-dependent effects can also be prevented, which is particularly beneficial for patients with impaired organ function and reduced clearance [18,19,20].

Data about practical implementation and derivation of therapy recommendations from TDM in routine care remain underrepresented compared to data on more theoretical pharmacokinetic approaches. However, this poses a particular challenge, as measured plasma concentrations should be translated into specific dosage optimizations that are tailored to the patient’s needs, but also pragmatic enough to be implemented under routine conditions. Interprofessional cooperation including clinical pharmacy services and algorithm-based recommendations, which also form the basis for computer-based solutions [21], could close the gap of transferring piperacillin/tazobactam TDM results into routine care.

We aimed therefore to investigate whether a TDM- and algorithm-based clinical pharmacy service increased the number of appropriately dosed patients in routine practice comparing their course of treatment. We assessed the attainment of the target piperacillin plasma concentrations.

## 2. Materials and Methods

*Patients and setting*: The study was performed in inpatients at the Bundeswehr Hospital in Hamburg (300 beds), a medium-sized standard healthcare provider offering care for military (20%) and civilian (80%) patients. We analyzed piperacillin plasma concentrations in patients hospitalized in the interdisciplinary intensive-care unit of this hospital. We performed this study as a retrospective analysis within the legal framework for quality assurance. In this context, we deliberately chose not to perform a prospective comparative intervention study, particularly due to the significantly greater legal requirements.

*Study design*: We performed a monocentric retrospective analysis of routine data after implementing a newly designed clinical pharmacy service.

*Study protocol*: The investigation consisted of the following three parts. First, a clinical pharmacy service was newly designed.

Second, an evaluation of the clinical pharmacy service was performed by verifying whether the target plasma concentrations of piperacillin were achieved. Third, intensive-care digital patient records and the pharmacy documentation were investigated for potential reasons for inappropriate plasma concentrations in (t4).

(i) The clinical pharmacy service consisted of the following two parts:
  (a) A new TDM analytic was developed and validated to analyze piperacillin blood concentrations.
  (b) An interprofessional expert panel consisting of physicians and pharmacists designed a dose adjustment algorithm based on the measured piperacillin plasma concentrations in intensive-care patients. This algorithm was intended to facilitate dose adjustments based on routine TDM and increase the acceptance of dosage recommendations given by clinical pharmacists to treating physicians.
(ii) The effects of the piperacillin algorithm- and TDM-based clinical pharmacy service in the time course of routine treatment of intensive-care patients were analyzed. For this purpose, we investigated the extent to which MIC-related target blood concentrations of piperacillin were attained under routine conditions.

We included retrospective data from a time frame of one year (1 September 2023 to 31 August 2024) after the clinical pharmacy service had been implemented. Data from patients who had been treated with piperacillin/tazobactam were included in the analysis. Additional inclusion criteria specified that data on the achievement of the target blood concentration during an intensive-care unit stay had to be available at least on the first working day after starting treatment. TDM measurements were performed only on working days (Monday to Friday). The time points t1, t2, t3, and t4 represent the first, second, third, and fourth working day after piperacillin/tazobactam therapy initiation, respectively. Since our intention was to analyze routine conditions, we actually evaluated, unlike a purely kinetic approach, only those workdays on which a TDM was performed, as recommendations were only made for those days. Weekend days were therefore not counted as measurement days. If therapy was initiated on Friday, t1 would be measured on the following Monday.

Data from other antibiotic regimens were excluded (even if TDM had been performed for them, such as for meropenem). Treatments outside the intensive-care unit were excluded.

*(i.a) Clinical pharmacy service: Therapeutic drug monitoring (TDM) analytic*: After receiving blood samples from routine intensive-care patients, they were processed by a validated method. At first the samples were centrifuged and the proteins were precipitated by using a mixture of methanol/acetonitrile. After performing another centrifugation, the samples were diluted with water and transferred into the high-performance liquid chromatography (HPLC). Phosphoric acid was used as the mobile phase with a gradual proportion of acetonitrile. The gradual proportion of acetonitrile over time was as follows (time—acetonitrile): 0 min—4%, 3.9 min—11%, 5.1 min—21%, 7.0 min—28%, 9.5 min—31.3%, 11.0 min—47%, and 12.0 min—4%.

For each day a corresponding analytic quality control (QC) was used before and after the samples. Calibration was performed at monthly intervals. Flow rate: 2 mL/min, pressure limit: 400 bar, injection volume: 20 µL, injection with needle wash: via flush port, sample tray temperature: 5 °C, HPLC: Agilent HPLC (Agilent Technologies Deutschland GmbH, 76337 Waldbronn, Germany) with 1260 Infinity High Performance Degasser G1379A, 1110 Infinity Binary Pump G1312A with piston backflush, 1260 Infinity Sample Injector G7129A, 1100 Column Oven G1316A (CS - Chromatography Service GmbH, 52379 Langerwehe, Germany) was used. MultoHigh 100 RP-8 (5 μm), 3 mm × 125 mm or comparable, Pre-column: suitable for the HPLC column, e.g., pre-column cartridge 10 × 3 packed with MultoHigh 100 RP 8, 5 μm (Art. No.: 7.5585306-1124), and column temperature: 30 ± 0.8 °C. Subsequently, an identity and quantitative determination was performed using a UV/VIS detector (Agilent 1260 Infinity Diodenarray-Detektor G7117C; Agilent Technologies, Santa Clara, CA, USA within the wavelength range of 240 nm and retention time of approximately 8.7–9.2 min. DAD detector: peak width: >0.025 min, stop time: as pump/injector, store: all in peak, range: 200–600 nm, Step: 4 nm, threshold: 1 mAU, auto-balance: pre-run, and bandwidth: 4.0 nm.

Piperacillin was verified for the 4–200 µg/mL range.

*Internal and external standard:* A 6-point calibration using cefotaxime as an internal standard (ISTD) was originally planned. Preliminary studies showed that the addition of the internal standard did not improve accuracy or precision. Therefore, deviating from the plan, the analysis was performed without an internal standard. In routine practice, quantification was carried out via a 1-point calibration using an external standard in porcine serum (piperacillin calibration level: 100 µg/mL).

Precision: The target was set internally as follows: relative standard deviation (RSD) or coefficient of variation (CV) < 6%. There was an analysis of two quality control samples (QC A and QC B), prepared from finished pharmaceutical products in porcine serum (*n* = 10). Result (*n* = 10): QC A piperacillin target: 20 µg/mL. Measured value: 18.69 µg/mL, CV = 2.33%. QC B piperacillin target: 60 µg/mL. Measured value: 56.83 µg/mL, CV = 2.02%. Result after excluding individual outliers (z > 2.5, *n* = 9). QC A measured value: 18.84 µg/mL, CV = 0.49%. QC B measured value: 57.20 µg/mL, CV = 0.45%.

*Accuracy:* Requirement: Deviation of measured value from target value < 12% for piperacillin. Comparison of 6-point vs. 1-point calibration (*n* = 3). Six-point calibration: deviation for piperacillin 8.5–10.3%. One-point calibration: deviation for piperacillin 0.5–0.7%.

*External accuracy:* Successful participation in proficiency testing schemes (RfB AK 04/22 and AK 02/23).

*Linearity:* Evaluation based on 3 independent calibration series, each with 6 calibration points. Correlation coefficients r ≥ 0.99989 (requirement r ≥ 0.999). Limit of quantitation (LOQ) was calculated from the standard deviation of the regression line and the slope. Calculated mean value: LOQ 2.6 µg/mL; established value for routine use: 4 µg/mL (precisely meets the requirement of 0.5 (minimum inhibitory concentration), ensuring reliable coverage of trough levels during extended bolus administration or continuous infusion).

Substances that may co-elute with piperacillin but are negligible under the selected analytical conditions: ampicillin, carbamazepine, fentanyl, isosorbidedinitrate, lamotrigine, nitrendipine, flucloxacillin, and xipamide.

Drugs demonstrated not to interfere with the piperacillin analysis: acetylsalicylic acid, allopurinol, argatroban, baclofen, bisoprolol, budesonide (inhaled), caspofungin, cefuroxime, chlorprothixene, colecalciferol, ciprofloxacin, cisatracuriumbesilate, citalopram/escitalopram, clindamycin, clonidine, co-trimoxazole, daptomycin, dexketoprofen, dexmedetomidine, digitoxin, dobutamine, enalapril, erythromycin, fluvastatin, furosemide, haloperidol, heparin, hydrochlorothiazide, hydrocortisone, insulin (actrapid), ramipril, reproterol, salbutamol (inhaled), simethicone, simvastatin, sitagliptin, spironolactone, sufentanil, tetrahydrocannabinol, ticagrelor, tobramycin, hydrochlorothiazide, hydrocortisone, insulin (actrapid), ipratropiumbromide (inhaled), levodopa/benserazide, levofloxacin, lorazepam, lormetazepam, L-thyroxine, glyceryltrinitrate, metoclopramide, metoprolol, metronidazole, midazolam, mirtazapine, morphine, N-acetylcysteine, naloxone, noradrenaline/norepinephrine, omeprazole, opipramol, ornithineaspartate, pantoprazole, piritramide, pramipexole, prasugrel, prednisolone, propofol, propranolol, ramipril, reproterol, salbutamol (inhaled), simethicone, simvastatin, sitagliptin, spironolactone, sufentanil, tetrahydrocannabinol, ticagrelor, tobramycin (inhaled), torasemide, urapidil, valsartan, vancomycin, and vitamins.

*(i.b) Clinical pharmacy service: Algorithm to recommend piperacillin/tazobactam dosing:* An interprofessional expert panel of physicians and pharmacists collaboratively developed an algorithm to determine the appropriate dosing of piperacillin/tazobactam based on piperacillin plasma concentrations received from the TDM. Furthermore, the target piperacillin plasma concentration ranges were derived from the bacilli EUCAST ECOFFs. Evidence to support a clear target is not documented in the existing literature. Based on the PK/PD model of β-lactam-antibiotics (t > MIC), potential mutant selection window and low, but dose-dependent, toxicity, a target range of 2–4 × ECOFF MIC has been chosen [8,22]. Targeted blood concentration range was developed based on the bacilli EUCAST ECOFFs: *Pseudomonas*: EUCAST MIC [mg/L] Susceptible (S) ≤ 0.001 mg/L. An MIC breakpoint of S ≤ 0.001 mg/L is an arbitrary, “off-scale” breakpoint (corresponding to a zone diameter breakpoint of “S ≥ 50 mm”) which categorizes wild-type organisms (organisms without phenotypically detectable resistance mechanisms to the agent) as “Susceptible, increased exposure” (I). For these organism–agent combinations, never report “Susceptible, standard dosing regimen” (S). Resistant > 16 mg/L, Target TDM [mg/L]: Susceptible, increased exposure (I) 32–64 mg/L; *Enterobacterales*: EUCAST MIC [mg/L] S ≤ 8 mg/L, R > 8 mg/L, Target TDM [mg/L]: S 16–32 mg/L; Unknown: calculated: 32–64 mg/L.

**Figure 1:**
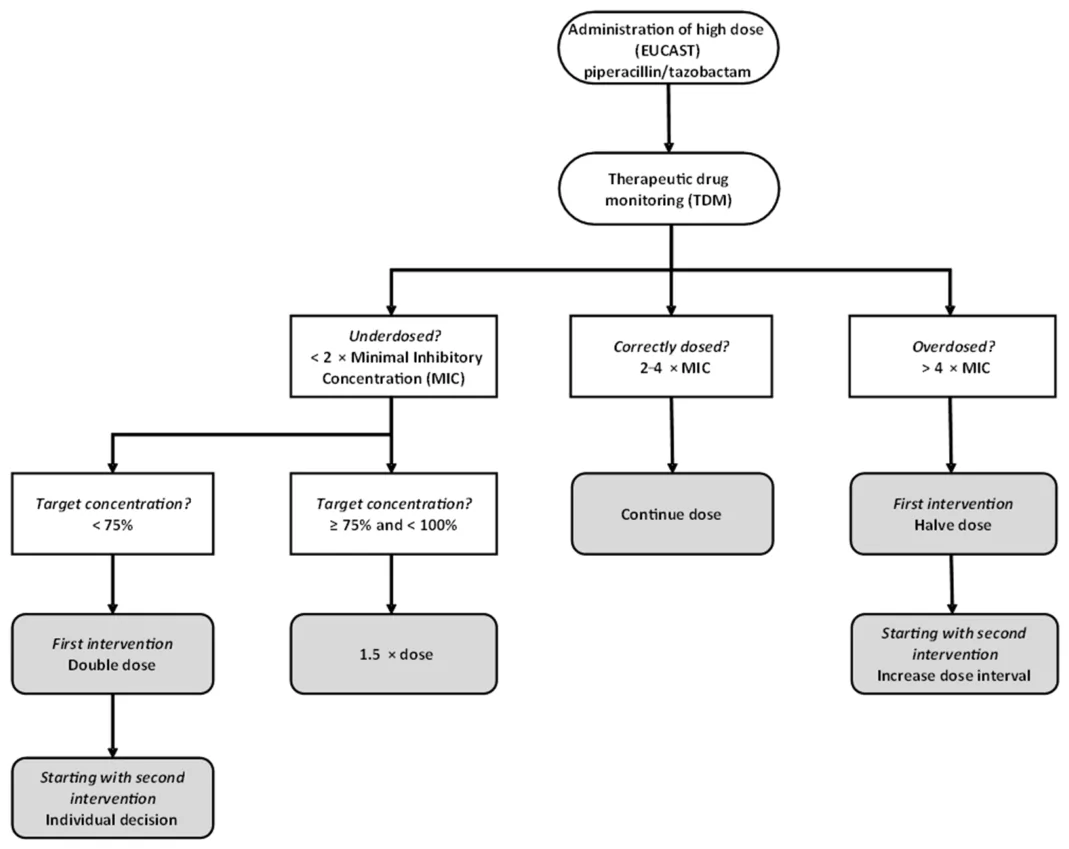
Algorithm for deciding recommendations for dose adjustments based on therapeutic drug monitoring (TDM) results. Piperacillin/tazobactam dosing is adjusted according to measured plasma concentrations and minimum inhibitory concentration (MIC) targets.

For piperacillin therapy, we aimed for 2 to 4 times the minimum inhibitory concentration (MIC) to ensure maximum bacterial eradication, compensate for differences in tissue anatomy, and prevent the development of resistance. As a beta-lactam antibiotic, piperacillin acts in a time-dependent manner. The concentration should remain above the inhibitory threshold for as long as possible. However, the concentration in infected tissue (e.g., lungs, abdomen) is frequently lower than in the blood. A serum concentration 2 to 4 times the MIC therefore guarantees effective concentrations at the site of infection even outside the blood. In intensive-care patients with severe infections or those with sepsis, the volume of distribution can additionally be increased; buffer zones above the MIC prevent therapeutic failure due to dilution effects. For safety and resistance prevention including prevention of mutations higher drug concentrations also kill less susceptible subpopulations of bacteria. The target range is usually between 32 and 64 mg/L (corresponding to 2 to 4 times the threshold value) but remains well below toxic levels to avoid side effects such as neurotoxicity. The algorithm used at the time of this analysis is shown in Figure 1.

Piperacillin/tazobactam routine treatment: Every patient was given a loading dose of 4.5 g piperacillin/tazobactam, followed by an extended 4.5 g piperacillin/tazobactam (over 4 h) every 6 h. The procedure was not modified for patients with impaired renal function or renal replacement therapy. Instead, we adjusted for the measured blood concentrations, which would also have been influenced by renal function.

TDM at the patient level: After introducing piperacillin TDM for all respective intensive-care patients receiving piperacillin/tazobactam a trough concentration was sampled in the morning. In order to ensure accurate and standardized trough-level sampling, the morning dose was always administered at the same time. Plasma levels were to be measured in the patient immediately before the next dose was administered.

Performing the TDM- and algorithm-based recommendation: The recommendations to the treating physician were based on the TDM results and the algorithm shown in Figure 1. After analyzing the sample, the results were verified for plausibility by a clinical pharmacist. The results and the dose adjustment based on the algorithm were conveyed by phone from a clinical pharmacist to a senior physician.

If a pathogen was detected (indication for targeted instead of calculated therapy) and tested susceptible during the intensive-care unit stay, the target concentration was adjusted accordingly to the predefined concentrations. In case microbiological results were already available, demonstrating the absence of *Pseudomons spec*., but no antimicrobial susceptibility testing (AST) was yet available, the target concentration was not adjusted, but falling 10% below the high (*Pseudomonas spec.*) concentration was accepted. In addition, a 10% exceeding of the target concentration was tolerated. The rationale for this was the risk that halving the dose at borderline values could result in falling below the target concentration and surpassing the range was considered less critical than undershooting the target concentration.

The final dose adjustment was always a decision by the treating physician based on the TDM, patient’s condition and microbiological results. The algorithm underlying clinical pharmacy services did not consider renal function as a separate factor, since we used the individual plasma concentration, which generally already accounted for limitations in all elimination processes. It should be noted, however, that we did not verify the renal steady state in advance.

*(ii) Evaluation: Routine piperacillin/tazobactam treatment:* The impact of the clinical pharmacy service was examined on the treatment of intensive-care patients in terms of achieving target concentrations of piperacillin in the blood relative to MIC. This was assessed at t1, t2, t3, and t4 (1–4 working days after piperacillin/tazobactam therapy was started). The data were summarized using Microsoft Excel (for Windows 11) and evaluated descriptively with regard to the frequency of the questions. Absolute and relative frequencies are given, as appropriate. Patients with multiple intensive-care unit admissions during the evaluation period were analyzed only for their first hospitalization. All patient samples were analyzed to determine whether they had reached the predefined (and, if necessary, adjusted to the treatment situation, e.g., targeted instead of calculated therapy) target plasma concentration range of piperacillin.

If values for t1 were missing, the corresponding data were not included. If values for t2–4 were missing, this was documented. Additionally, we investigated reasons for inappropriate plasma concentrations at the end of the evaluation (t4).

*Statistics:* We performed a McNemar test (available from https://www.graphpad.com/quickcalcs/mcnemar1/, accessed on 10 September 2025) to assess the number of patients with too low, appropriate, and too high piperacillin plasma concentration. We used a McNemar test to compare pairs of plasma samples, which were to be matched, because we were interested in these individual comparisons rather than just an overall comparison of the four groups. For the comparison using a McNemar test, the pairs for which values were still available at the later measurement time points (t2–t4) were included in the pairwise analysis. Since three comparisons were performed (t1 vs. t2; t1 vs. t3; and t1 vs. t4), a Bonferroni correction was applied, and *p* ≤ 0.0167 was considered statistically significant.

## 3. Results

### 3.1. Patient Characteristics

**Figure 2:**
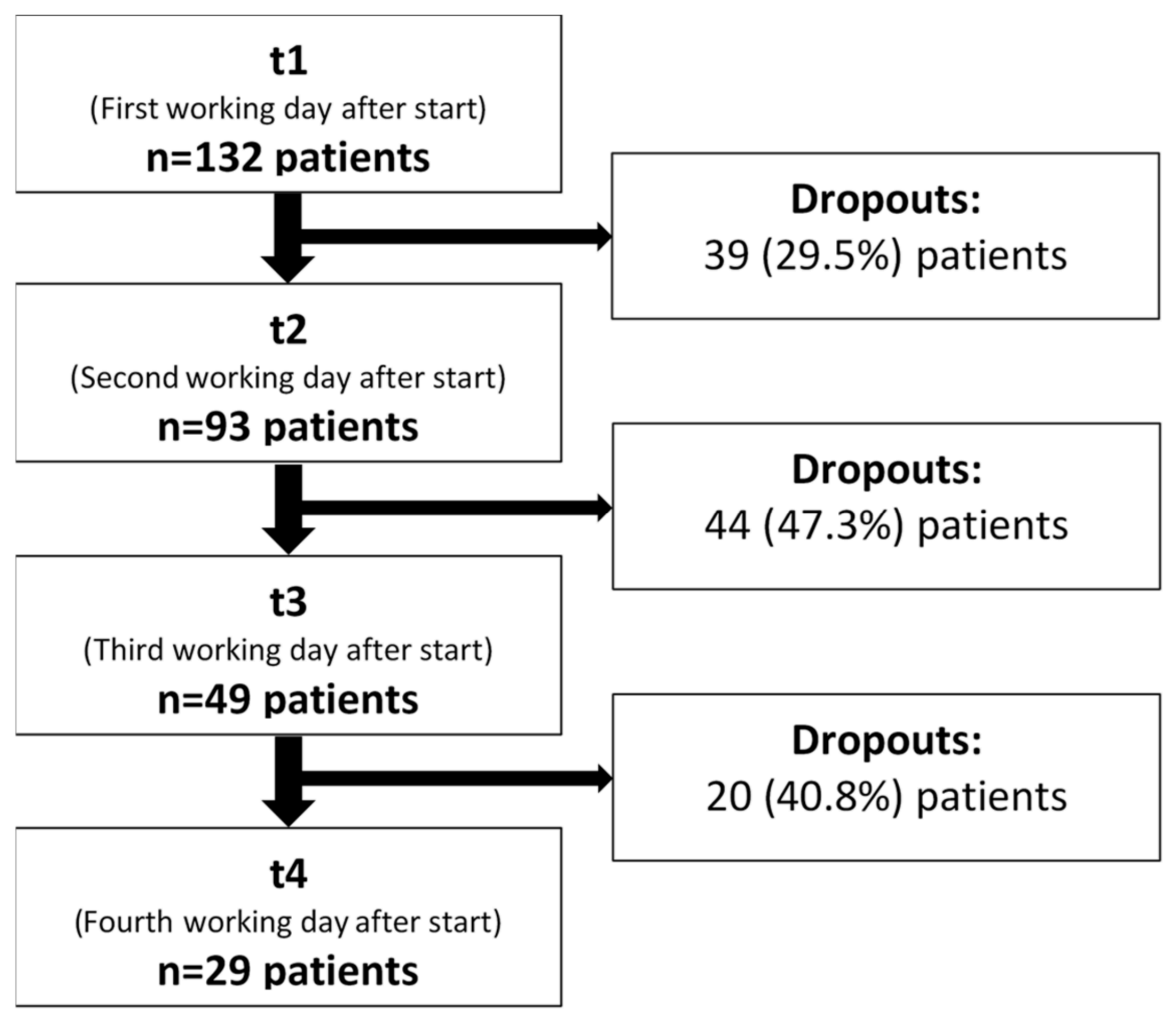
Flowchart of the analyzed patient data at time points t1–t4.

As shown in Table 1, a total of 132 patients (including 47 women, 36%) with a median age of 78 (min. 25–max. 96) years underwent TDM of piperacillin, with sepsis or septic shock (43%) as the most common indication. *n* = 4 samples were excluded from analysis due to equipment malfunction or improper sample collection. During this study, 306 samples in total were analyzed. Thereof, 285 samples were empirical (t1: 129, t2: 86, t3: 42, and t4: 25) and 21 (t1:3, t2:7, t3:7, and t4:4) targeted therapy. The main reasons for the decreasing number in TDM samples were transfer to another ward, switch of the antibiotic, or clinical cure. Figure 2 shows the flowchart of the analyzed patient data at time points t1–t4.

### 3.2. Attainment of the Target Piperacillin Plasma Concentrations

As presented in Table 2, from a total of *n* = 132 patients in (t1), 19% had “too low piperacillin concentrations”, 20% had “appropriate concentrations”, and 61% had “too high concentrations”. After recommendations were given the corresponding numbers from *n* = 93 patients remaining in (t2) were 16%, 35%, and 48%, from *n* = 49 patients in (t3) 8%, 53%, and 39% and from *n* = 29 patients in (t4) 38%, 45%, and 17%.

### 3.3. Recommendations to Optimize Piperacillin/Tazobactam Dosing

As presented in Table 3, the main recommendations given according to the predefined algorithm were “dose reduction of piperacillin/tazobactam” in (t1) and “no dose adjustment” in (t2), (t3), and (t4).

### 3.4. Reasons for Inappropriate Plasma Concentrations at the End of the Evaluation (t4)

In *n* = 11 patients who had “too low concentrations” at t4, the value fell only 10% below the threshold but no error or underlying cause was apparent (*n* = 4); the value was below the threshold possibly due to improved renal elimination (*n* = 3), the measurement was taken despite piperacillin having already been discontinued (*n* = 1), adjustment of the time interval led to a missed dose (*n* = 1), the value was below the threshold due to dialysis (*n* = 1), and there was an excessive reduction (*n* = 1).

## 4. Discussion

Over a period of one year, we found that inappropriate plasma concentrations of piperacillin were surprisingly common. Only one in five piperacillin patients was already within the target plasma concentrations at the first measurement after initiating therapy. “Too high concentrations” occurred frequently (61%) in the beginning of piperacillin/tazobactam therapy (t1, i.e., one working day after therapy was started). This suggests that rigid dosing, as initially performed, frequently does not result in a piperacillin concentration within the target concentration. By this, our results from routine care also show that there is a tendency for overdosing at the beginning.

While this seems understandable given the low risk of adverse drug reactions caused by piperacillin/tazobactam, it should be considered that excessively high plasma concentrations also pose a risk, e.g., for neurological and nephrological complications. Compared with published studies, the distribution of plasma concentration in our study appears notable. Proportionally, we observed fewer underdosing and a higher rate of overdosing. This is likely to be caused by different target concentrations, lower prescribed doses, lack of loading dose or the exclusion of patients with renal impairment [5,18,20,23].

Based on TDM and algorithm-based recommendations from the clinical pharmacy service, we succeeded particularly in avoiding overdoses (t1 vs. t2; t1 vs. t3; and t1 vs. t4). Underdosing was not influenced according to all tested comparisons. Correct dosing was significantly improved in only one comparison (t1 vs. t3). We made a conscious decision to test all three categories (i.e., underdosed, correctly dosed, and overdosed) separately, as each requires different clinical decisions and therefore warrants its own evaluation. The focus is thus not merely on correct dosing, but specifically on whether underdosing or overdosing occurs and whether these issues are resolved by the clinical pharmacy service. Consequently, “dose reduction” was the most common recommendation. All given recommendations were adopted by treating physicians (100% acceptance) in the treatment protocol.

At the last measurement point (t4), for which only a relatively limited number of measurements were available, underdosing remains to be observed in relatively high number of patients. We therefore analyzed possible reasons to understand why misdosing was still observed in a relatively large proportion of patients. According to the intensive-care digital patient records and the pharmacy documentation, “restoration of organ excretion capacity” and “unintentional process deviation” were main possible reasons particularly for underdosing remaining at t4. However, it should be borne in mind that the number of patients who could be assessed at this final point in time was significantly reduced. This was presumably the case since patients could be transferred to peripheral facilities after their medical condition improved. Moreover, a withdrawal of the antibiotic due to therapeutic failure/toxicity can be another reason for dropouts at later time points. Additionally, high dropout rates can be explained in patients of an intensive-care unit with high mortality rates. We should therefore assume that there might be an accumulation of particularly critical patients at this time.

Renal function, measured by a GFR of 42.5 mL/min (Q25; Q75 22.8; 72.3 mL/min), indicates that numerous patients have severely impaired renal function (8% of our patients underwent renal replacement therapy). With a Q(0) value of 0.3 for piperacillin, a prolonged half-life can occur, particularly with very low clearance values.

However, it should be noted that, given the routine testing conditions, we could not definitively conclude that there was stable renal (dys)function. Therefore, not only is deterioration in renal function possible but an improvement is as well, which could also very well explain the high number of patients with too low piperacillin concentrations at the end of t4.

In [24], the authors reported the implementation of a TDM program for beta-lactam antibiotics in a large academic medical center with a rate of 15% treated patients with piperacillin. The authors conclude that beta-lactam TDM resulted in dose adjustments in 25% of patients and that standard antibiotic dosing did not achieve optimal PK/PD targets in all patients. These data, which were obtained using a 4 × 4.5 g dose of piperacillin administered as a short-term infusion, support our findings that the initial dose generally does not achieve the desired target concentrations and therefore justify a TDM for dose individualization. However, we deliberately also focused on the follow-up measurements, which showed that further follow-up measurements seem reasonable, since even then, deviations from target plasma concentrations still occur.

In a randomized multicenter controlled trial [25], adult patients with sepsis or septic shock were randomly assigned 1:1 to receive continuous infusion of piperacillin/tazobactam with dosing guided by daily TDM of piperacillin/tazobactam or continuous infusion with a fixed dose (13.5 g/24 h if eGFR ≥ 20 mL/min). Although patients in the TDM group achieved target plasma concentrations significantly more frequently, this had no significant impact on the SOFA score. Furthermore, any benefits on mortality and clinical and microbiological aspects were narrowly missed [23]. This also highlights the limitations of TDM. It can only serve as an aid. A cleverly structured dose adjustment is at least equally important. As is well known, measured values alone do not result in better therapy.

In [26], the study used four measurement time points for critically ill patients receiving piperacillin–tazobactam therapy, just as in our study. While the authors consider beta-lactams to be appropriate therapies, they conclude that current dosing practices for intensive-care patients may be suboptimal [27]. This also underscores the need for intensive TDM-supported therapy monitoring.

With regard to the variability of influencing factors, ref. [26] found that in univariable and multivariable logistic regression, body weight and estimated glomerular filtration rate were negatively associated with goal achievement.

Another study [27] showed that, in addition to dose optimization through TDM, other aspects should also be considered in pharmaceutical counseling. This particularly applies to intensive-care patients with multiple parenteral therapies, including incompatibilities in the delivery systems and, if applicable, mixed bags.

In clinical practice, the use of the algorithm and the interprofessional involvement of clinical pharmacists has shown to be a possibly valuable tool in deciding on dose adjustments. In the future, this approach could therefore be used in other clinical situations for faster, reproducible decision-making. However, the experience from this study has also shown that ongoing TDM with resulting recommendations is useful, as initial interventions cannot solve all problems in the long term. As demonstrated in this study, individual changes in the patient’s pharmacokinetics should be continuously taken into account. Therefore, future work may consider incorporating potential changes in clearance (recovery renal clearance) as a factor into the algorithm. Additional staff training, which was not the subject of this study, could further increase understanding and acceptance of recommendations. In addition, evidence-based target concentrations should always be questioned, as shown by recent studies that suggest that setting more aggressive target concentrations could offer potential benefits [28].

Our findings could have several potential clinical implications for intensive-care practice. First, the high rate of initial too high concentrations (61%) suggests that standard dosing regimens (4.5 g q6h with extended infusion) frequently exceed target concentrations, whilst this is being accepted due to the necessity of avoiding critical underdosing; dose adjustments are inevitable to avoid potentially unnecessary risks such as neurotoxicity. Second, the dynamic nature of intensive-care patient pharmacokinetics, evidenced by the 38% too low concentrations rate at t4 despite earlier optimization, highlights the need for repeated TDM measurements rather than single-point assessments.

### Limitations and Sources of Potential Bias and Confounders

This study has the following limitations:

First, our study focused exclusively on TDM-related dosing recommendations and did not assess clinical outcomes such as mortality, length of stay in intensive-care units, or microbiological cure rates. While improved target attainment is a validated surrogate endpoint, the TARGET trial [17] indicated that better PK/PD target achievement does not automatically translate into improved mortality.

Second, due to the routine protocol, standardized conditions, particularly steady-state conditions, could not be maintained with regard to both antibiotic dosing and renal function.

Third, with piperacillin/tazobactam a specific antibiotic was investigated in a narrowly defined patient group. Due to the limited patient size, we were unable to perform subgroup analyses to identify particular risk groups or potential confounder (e.g., renal function, age, disease severity or sex). Therefore, extrapolations to other drugs and patient population should be made with caution.

Fourth, calculated and targeted therapy were evaluated together. Naturally, different target concentrations were considered according to relevant guidelines. Nevertheless, it cannot be ruled out that there could be additional differences in the evaluation that were not taken into account in this assessment.

Fifth, although the procedure was tied to an algorithm and the clearly defined measured values were highly standardized, it cannot be completely ruled out that the positive results were also (at least in part) due the good personal cooperation within the interprofessional team. Finally, our study design does not allow us to definitively determine the reasons why target concentrations were partially not reached or exceeded. Even though we had exanimated possible reasons (such as recovery of elimination), other reasons (such as deliberate algorithm deviations) leave questions unanswered. Ultimately, the causes are likely to be multifactorial anyway.

Sixth, it can be considered a limitation that the data presented here were not compared with a control group from the period prior to the introduction of the clinical pharmacy service. Due to the lack of relevant retrospective data on therapeutic drug monitoring (TDM) in the absence of clinical pharmacy services, it was unfortunately not possible to analyze a historical control group. Only limited data regarding dosing regimens involving fixed-dose combinations would have been available for that period. However, the primary objective of this study was not to demonstrate the benefit of TDM-guided piperacillin dosing per se, but rather to illustrate the extent to which the associated clinical pharmacy service can actually lead to improvements for individual patients.

## 5. Conclusions

Our analysis indicated that inappropriate piperacillin plasma concentrations were common in routine clinical practice. A clinical pharmacy service based on TDM and an algorithm appeared to particularly decrease the number of patients receiving overdoses of piperacillin/tazobactam. Attending physicians implemented all of the service’s recommendations. The results suggest that the service significantly reduced the incidence of excessively high piperacillin concentrations in particular. Nevertheless, at the end of the evaluation period, a substantial number of patients still exhibited plasma concentrations that were too low. While the small sample size precludes the identification of a primary cause, a recovery of renal elimination could be a likely explanation for some of these patients.

## Figures and Tables

**Table 1:** Patient characteristics. BMI: body mass index; eGFR: estimated glomerular filtration rate. PCT: procalcitonin; SOFA-Score: sequential organ failure assessment score.

Parameter	Unit	Patients Female (*n* = 47)	Patients Male (*n* = 85)	Patients Total (*n* = 132)
Age [years]	Median	77	78.5	78
	(Q25; Q75)	(63; 85)	(66; 84)	(66; 84)
Death	*n*	9	14	23
	(percent)	(19%)	(16%)	(17%)
Weight [kg]	Median	70	81	80
	(Q25; Q75)	(59; 85)	(70; 91)	(65; 90)
BMI	Median	26.2	26.1	26.1
	(Q25; Q75)	(22.1; 32.4)	(22.9; 29.4)	(22.6; 29.6)
eGFR [ml/min]	Median	41.0	45.5	42.5
	(Q25; Q75)	(20.0; 68.5)	(26.5; 75.8)	(22.8;72.3)
SOFA score	Median	8	8	8
	(Q25; Q75)	(6; 10.3)	(6; 9.5)	(6; 10)
PCT [ng/mL]	Median	1.7	1.4	1.5
	(Q25; Q75)	(0.9; 7.9)	(0.4; 4.7)	(0.6; 6.4)
Renal replacement therapy	*n*	5	5	10
	(percent)	(11%)	(6%)	(8%)
Sepsis/septic shock	*n*	21	36	57
	(percent)	(45%)	(42%)	(43%)
Pulmonary focus	n	14	39	53
	(percent)	(31%)	(46%)	(40%)
Gastrointestinal focus	n	19	17	36
	(percent)	(40%)	(20%)	(27%)
Urogenital focus	*n*	9	18	27
	(percent)	(19%)	(21%)	(21%)
Unknown focus	n	6	14	20
	(percent)	(13%)	(16%)	(15%)
Bacteremia	*n*	4	2	6
	(percent)	(9%)	(2%)	(5%)
Dermal focus	*n*	3	1	4
	(percent)	(6%)	(1%)	(3%)
Prolonged prophylaxis	*n*	0	2	2
	(percent)	(0%)	(2%)	(2%)
Ear, nose, and throat focus	*n*	2	0	2
	(percent)	(4%)	(0%)	(2%)
Endocarditis	*n*	1	0	1
	(percent)	(2%)	(0%)	(1%)
Ostitis/Osteomyelitis	*n*	0	1	1
	(percent)	(0%)	(1%)	(1%)

**Table 2:** Piperacillin blood concentrations over treatment time. t1, t2, t3, and t4 (1–4 working days after piperacillin/tazobactam therapy was started). MIC: minimum inhibitory concentration depending on the specific targeted (including pathogen detection) or calculated therapy. * The comparison was performed using a McNemar test, in which the pairs for which values were still available at the later measurement time points (t2–t4) were included in the pairwise analysis. Since three comparisons were performed (t1 vs. t2; t1 vs. t3; t1 vs. t4), a Bonferroni correction was applied, and *p* ≤ 0.0167 was considered statistically significant. ** Due to rounding, the total comes to 99%.

Working Days After Piperacillin/ Tazobactam Therapy was Started	Underdosed (<2 × MIC)			Correctly Dosed (2–4 × MIC)			Overdosed (>4 × MIC)			Total (*n*)
	**[*n*]**	**[%]**	***p*** *	**[*n*]**	**[%]**	***p*** *	**[*n*]**	**[%]**	***p*** *	
t1	25	19%	-	27	20%	-	80	61%	-	132
t2 ** (*p*-value: t1 vs. t2)	15	16%	n.s.	33	35%	n.s.	45	48%	0.0080	93
t3 (*p*-value: t1 vs. t3)	4	8%	n.s.	26	53%	0.0013	19	39%	0.0056	49
t4 (*p*-value: t1 vs. t4)	11	38%	n.s.	13	45%	n.s.	5	17%	0.0095	29

**Table 3:** Recommendations according to the algorithm. * In-house transfer refers to transfer from intensive-care unit to general ward with discontinuation of therapeutic drug monitoring.

Working Days After Piperacillin/Tazobactam Therapy was Started	Dose Increased	No Dose Adjustment	Dose Reduced	Piperacillin/ Tazobactam Discontinued	In-House Transfer *	Total
t1	13 (10%)	36 (27%)	65 (49%)	7 (5%)	10 (8%)	132
t2	6 (6%)	40 (42%)	17 (18%)	14 (15%)	16 (17%)	93
t3	0 (0%)	30 (60%)	12 (24%)	6 (12%)	1 (2%)	49
t4	4 (14%)	10 (34%)	4 (14%)	6 (21%)	5 (17%)	29

## Data Availability

The data presented in this study are available on request from the corresponding author, insofar as this is justifiable for ethical and data protection reasons.
